# Probing Different Approaches in Ultraviolet Radiation Personal Dosimetry – Ball Sports and Visiting Parks

**DOI:** 10.3389/fpubh.2022.868853

**Published:** 2022-04-27

**Authors:** Timo Heepenstrick, Claudine Strehl, Marc Wittlich

**Affiliations:** ^1^Department Ergonomics, Physical Environmental Factors, Institute for Occupational Safety and Health of the German Social Accident Insurance, Sankt Augustin, Germany; ^2^Department Accident Prevention, Digitalisation – Technologies, Institute for Occupational Safety and Health of the German Social Accident Insurance, Sankt Augustin, Germany

**Keywords:** UV radiation, personal dosimetry, personal exposure, health prevention, exposure registry

## Abstract

Solar ultraviolet radiation (UVR) continues to be a decisive influencing factor for skin health. Besides acute damage (e.g. erythema), chronic light damage is of particular relevance. Skin cancer can develop on the basis of this light damage. Knowledge about irradiation is crucial for the choice of preventive measures, but has so far been incomplete in many occupational and leisure activities. Often a methodological problem in study design is the cause. Here we report on the clarification of two issues. First, further values are to be determined on the way to a comprehensive exposure register of leisure-related activities. Furthermore, it is to be determined to what extent the measurement setting can have an influence on the measurement campaigns. For long-term measurements, football referees were equipped with dosimeters over several months, selective measurements during visits to parks were carried out by on-site recruitment of test persons. It turned out that the choice of method also depends on the expected compliance of the test persons. Long-term measurements of specific activities such as playing football are particularly suitable for observing the course of UV exposure over the year and generating resilient mean values. Point measurements such as visits to parks can also do this if there are enough such events spread over the year. However, they are particularly suitable for such on-site campaigns, as they may be combined with awareness campaigns of the issue of skin cancer. They also allow many measurements to be taken at the same time in one place. Both playing football and visiting parks are associated with high levels of radiation, so specific prevention concepts need to be developed. We were able to determine that the sunburn dose for light skin types was reached or exceeded for both of the investigated activities.

## Introduction

Ultraviolet (UV) radiation has been known to be a complete human carcinogen for many years and was classified by the International Agency for Research on Cancer (IARC) in Group 1 (“carcinogenic to humans”) as early as 1992 (1). UV radiation has a broad spectrum of effects on the human body, both beneficial and harmful. UV radiation is essential for the production of vitamin D3 (2, 3) but causes short-term (e.g., sunburn) as well as long-term (e.g., skin aging) damage if the exposure is too high. Chronic light damage can result in skin cancer; this includes various entities with different causative mechanisms, but all directly related to UV exposure. Squamous cell carcinomas (SCC) and their precursors, actinic keratoses (AK), are caused by cumulative UV exposure (4), while basal cell carcinomas (BCC) are likely related to the intensity and duration (intermittency) of UV exposure (5). Statistically, these entities have a ratio of 4:1:10 (BCC, SCC, AK) (6–8).

People are permanently exposed to UV radiation, both in their leisure time and their work environment. The latter, in particular, can lead to extremely high levels of irradiation, which require special medical screening (9). As a rule, employees do not have the possibility to choose whether or not they are exposed but are forced to rely on preventive measures. Several papers have already dealt with this topic in the past (10–16). To ensure comprehensive protection against UV radiation, a holistic approach for prevention is of great importance. This, therefore, includes leisure time activities.

It is of great importance to know what the actual irradiation is in order to be able to assess the risk and implement the appropriate measures correctly. The use of standardized, suitable measurement technology significantly contributes to acquiring this knowledge. While polysulphone film dosimeters (PSF) or biological spore dosimeters were often used in earlier measurements of personal UV exposure (17–20), more recent studies focus on the use of electronic data logger dosimeters (10, 12, 21–23). Comparative studies have clearly shown the latter's advantages (24).

Many studies on individual leisure time activities and the corresponding UV dose already exist. An overview of these has been provided earlier (25), while the behavior of sunbathers was studied, for example, in detail (26). Three groups were identified: suntanned, non-suntanned and photosensitive individuals. The personal UV doses of the groups were 259 J/m^2^, 236 J/m^2^ and 204 J/m^2^, respectively, within a maximum measurement time of 134 min at noon. The ambient UV doses were also measured and averaged 1249 J/m^2^, 1202 J/m^2^, and 1121 J/m^2^, respectively. In a study by Sun et al. (27), measurements of UV exposure in the population were conducted in Australia. For this purpose, participants were asked to wear polysulphone film dosimeters on their left wrist for 10 days. This took place at four locations (Townsville, Brisbane, Canberra and Hobart) and at four seasons. The average values of the personal UV dose per day range from 30 J/m^2^ to 200 J/m^2^ in the different seasons and locations. There is a study from 2007 for the activity “playing football” (28). This study involved fitting dosimeters to the faces of schoolchildren in Australia and having them play games of basketball and football for 1 hour. This resulted in average UV exposure of 99 J/m^2^ to 140 J/m^2^.

Several other studies have focused on determining the UV exposure doses received during specific activities like cycling, jogging and hiking (16, 17, 29–34). They differ mainly in the selection of the participant collective, the measurement technology used, the duration of the measurement, and the selection of the activities studied. Intercomparison is possible, but with certain assumptions regarding systematic deviations (24) and time and geographical particularities. Our study was designed in a way to cancel out intrasystematic deviations regarding the measurement technology and statistical uncertainties due to small sample numbers.

This study addresses two questions. The first is to determine additional values as part of a comprehensive exposure register of leisure-related activities. The second is to determine how the measurement setting can influence the measurement campaign itself. Basically, different approaches for obtaining data according to specific activities may be of use: either the participants are equipped with dosimeters over a long period of time and measurements are performed during a specific activity, or alternatively, an activity can be measured specifically on individual days with a large number of test persons simultaneously. For leisure time exposure, for example, public or sporting events are suitable. We chose football and visiting parks/recreational trips as a good way of addressing the research questions.

## Materials & Methods

### Test Person Collectives and Measurement Locations

In the run-up to the measurements, we consciously opted to select two activities that, in our view, are particularly appropriate and provide an excellent example of the leisure behavior of the “general public.” These activities are football and visits to parks. Football was chosen because it is a ball sport that is widely played and, in a broader sense, can also be used as a symbol for other ball sports. Furthermore, this activity is practiced all year round. The German Football Association (DFB) was called on to help recruit participants. Under FIFA and DFB rules, there are strict regulations regarding items worn on the body during training or matches. Consequently, it was not possible to equip players themselves with dosimeters. This regulation does not apply to the same extent to referees or coaches of the amateur leagues, so these two groups were asked to wear the dosimeters during the course of our measurements. Referees, in particular, move around the pitch in the same way as players. The movement pattern is also comparable to coaches during training. Furthermore, measurements could be taken at some special events such as tournaments, where several short matches took place on 1 day. The measurements took place in 2018 and 2019, from May to October in each instance. The possible measurement times were 4:30 p.m. to 9:00 p.m. from Monday to Friday, and 10:00 a.m. to 9:00 p.m. on Saturday and Sunday. In total, 33 people participated actively (16 in 2018 and 17 in 2019).

For the centralized single-day measurements at a large event, covering the leisure time activity “visiting parks”, the aim was to equip as many volunteers with dosimeters as possible at the same time and for a whole day. The measurements were performed on specific days during a federal garden show (Bundesgartenschau) that took place from April to October 2019 in Heilbronn, Germany. In total, measurements were done on 5 days during this period. More precisely, the measurements each took place on 1 day in April, June, and August and on 2 consecutive days in September (17/04, 18/06, 27/08, 20/09, 21/09). A prerequisite for the measurements was to have stable and dry weather conditions. For each measurement day, 15 dosimeters were available that were randomly distributed to interested visitors of the garden exhibition. The participants were recruited after entering the event and returned the dosimeters before leaving. On account of this, the measurement duration varied between the volunteers and the measurement days. Ambient UV exposure was also recorded at the same time. Hence, exposure conditions could be calculated from the ratio between ambient and personal UV exposure (18). The participants were told to behave as they would normally do, but an influence of the behavior cannot be completely ruled out (Hawthorne effect). Theoretically, two opposing effects are conceivable: First, people may spend more time in the sun than they normally do, second, people may seek more shade than they normally do. Both would give a footprint in the gyroscope data by time intervals of resting with simultaneously high or very low exposure data – both could not be detected. From our experience with 1,000 participants in another UV study, we received numerous proves that participants “forgot” the dosimeter after a certain time while wearing them; this might be attributed to the location where the device was worn in combination with its light weight.

### Exposure Measurement Technique

The participating volunteers were equipped with the GENESIS-UV measurement technology, consisting of an electronic dosimeter for measuring personal UV exposure and a tablet PC to regularly transfer the measured data to the Institute for Occupational Safety and Health. During the “visiting parks” activity, the researchers conducted the latter task immediately after the measurements were taken so that no other technology needed to be given to the participants.

The personal UV exposure was measured via electronic dosimeters of the type X2012-10 V3 from Gigahertz Optik (Türkenfeld, Germany). Our GENESIS-UV system for decentral UV exposure measurements has been described earlier (10). In brief, the dosimeters use two separate UV sensors (one for UV-A and one for UV-B/C) to measure the UV radiation erythema-weighted to a maximum resolution of 1 s. Erythema-weighting is achieved by built-in filters which reflect the spectral sensitivity of the skin to develop erythema. The erythema action spectrum S_er_ has been defined by the International Commission in Illumination (CIE) and is anchored in international standards (35). This provides detailed information about the exposure. Any average values for any condensed time interval can be calculated based on the per-second values. For reasons of checking data reliability, the dosimeters contain gyroscopes. By analyzing the accelerometer data from the gyroscope, information can be obtained to determine whether the dosimeters were accelerated or were resting. This can be a way of checking whether or not the dosimeters were worn properly while the measurements were taken. The devices were attached to the left upper arm via a tissue strap.

### Stationary Measurements and Data Analysis

An additional dosimeter was used to record the ambient UV radiation for the measurements taken during the visits of the garden exhibition. This dosimeter was mounted horizontally on a pedestal in the park, free from shading.

Stationary measurements are particularly important for measurements on a few individual days in order to put the personal measurements into an overall context. In contrast to spore or polysulphone film dosimeters, the choice of electronic dosimeters also allows the measurement and resolution of a temporal sequence over the course of the day. This can significantly help determine whether people's behavior changes at times of exceptionally high UV exposure (such as mid-day).

Since the dosimeters have a cosine dependence for detecting UV radiation, the resulting curve of ambient radiation is a combination of the radiation from the sun and the cosine of the angle between the sun and the detector normals.

In order to achieve intercomparability of the measurement results, the ratio between personal (UV_pers_) and ambient (UV_amb_, incoming radiation on a flat horizontal surface over the same exposure time period of personal exposure) UV exposure was calculated (exposure ratio to ambient, ERTA) (18). The ERTA is expressed as a percentage and calculated as follows: $\frac{UV_{pers}}{UV_{amb}}*100\%$.

This ratio has previously been estimated to be approximately 3% to 5% as an annual average and about 30% while being outside during the day (36). As described in their publication, the dosimeter was worn on the forehead, which is comparable to our positioning of the dosimeter on the left upper arm (37). Accordingly, our measured values can be used directly to calculate the ERTA without positional conversion.

The UV radiation data and the motion data of the accelerometer were evaluated in relation to each other for data analysis. Any areas in the data that did not show simultaneous movement were removed. The assumption could be made that the dosimeter was not worn on the person at these times. A procedure was also used to recalibrate the data with respect to dosimeter calibration, longitude-time correction and similar factors. After processing the raw data, the data available at one-second intervals were combined into intervals. Every 60-s measurement interval was totalled to get a minute value. Incomplete minute intervals at the beginning and end of a measurement series were ignored.

### Comparison With Yearly and Daily Variations in the Solar Irradiance

Global radiation is subject to both an annual and a daily cycle. It makes sense to analyze the data acquired in comparison to this. The distribution of UV irradiation over the year or over the day from an earlier publication is used for this purpose (11). The values are also related to the month of the solar maximum in June in order to make a relative comparison of the months easier. Given that the reference to the diurnal patterns will only serve as an illustration, this conversion is not necessary. Supplementary Figure 1 shows the corresponding curves and indicates the corresponding values under the histograms.

The measurements of the measurement campaign do not span the entire year, so the missing period must be extrapolated based on the seasonal factors. Assuming that the investigated activities were carried out in the same way in the missing period, a linear extrapolation can be carried out for the required periods.

## Results

### Long-Period Measurements: Football Games

**Figure 1:**
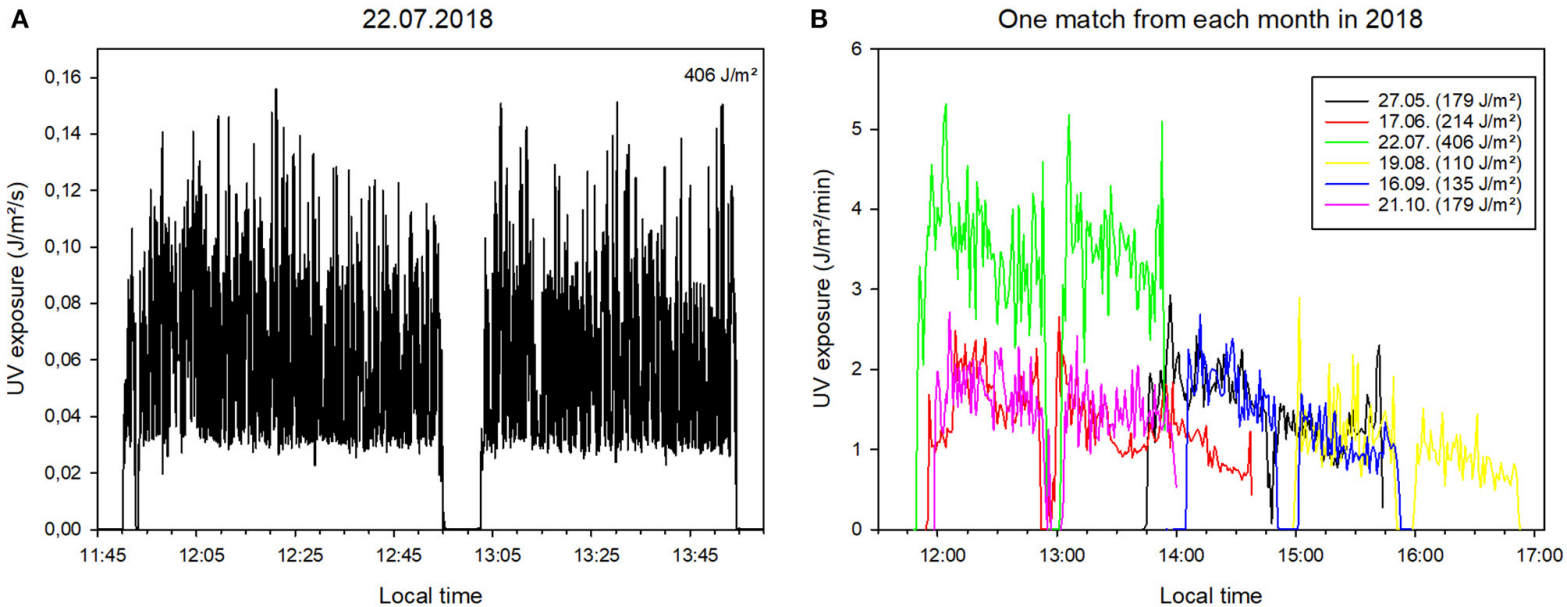
Representation of individual measurements of UV exposure during football matches. **(A)** UV exposure over time for a selected football match on 22/07/2018. Data is given in 1-s intervals. **(B)** Direct comparison of personal UV exposure acquired during football matches at different times of the day and during the year. Data is given in 1-min intervals for clarity.

In total, the football referees accumulated 35,372 min of measurement on 237 measurement days. Figure 1A shows the UV exposure for a football game in the month of July. This shows the two half-times with a break, which was obviously spent indoors. This behavior can be observed in most measurements for “playing football.” The course of the measured values provides detailed information on personal UV exposure during a football game. A clear distinction between active and resting (pauses) phases can be seen when taking the data from the accelerometer into account. Resting phases are identifiable by values of |a| around 1, which corresponds to the accelerometer experiencing only earth's gravity. The highest UV exposure dose (406 J/m^2^, i.e. 4.06 SED; 1 SED equals 100 J/m^2^ erythemal weighted irradiance) was measured during a football game taking place in July around noon in the early afternoon. By combining these data of several matches taking place in different months to a single plot, the differences in the data course and in UV exposure can be distinguished more clearly (Figure 1B).

In direct comparison to a football match taking place in June at approximately the same time and with comparable duration with a total UV exposure dose of 214 J/m^2^, the UV exposure is almost 2-fold higher in July. Comparable results for the total UV exposure dose can be seen for football matches in May in the afternoon (179 J/m^2^) and October beginning from noon to the early afternoon (179 J/m^2^).

**Figure 2:**
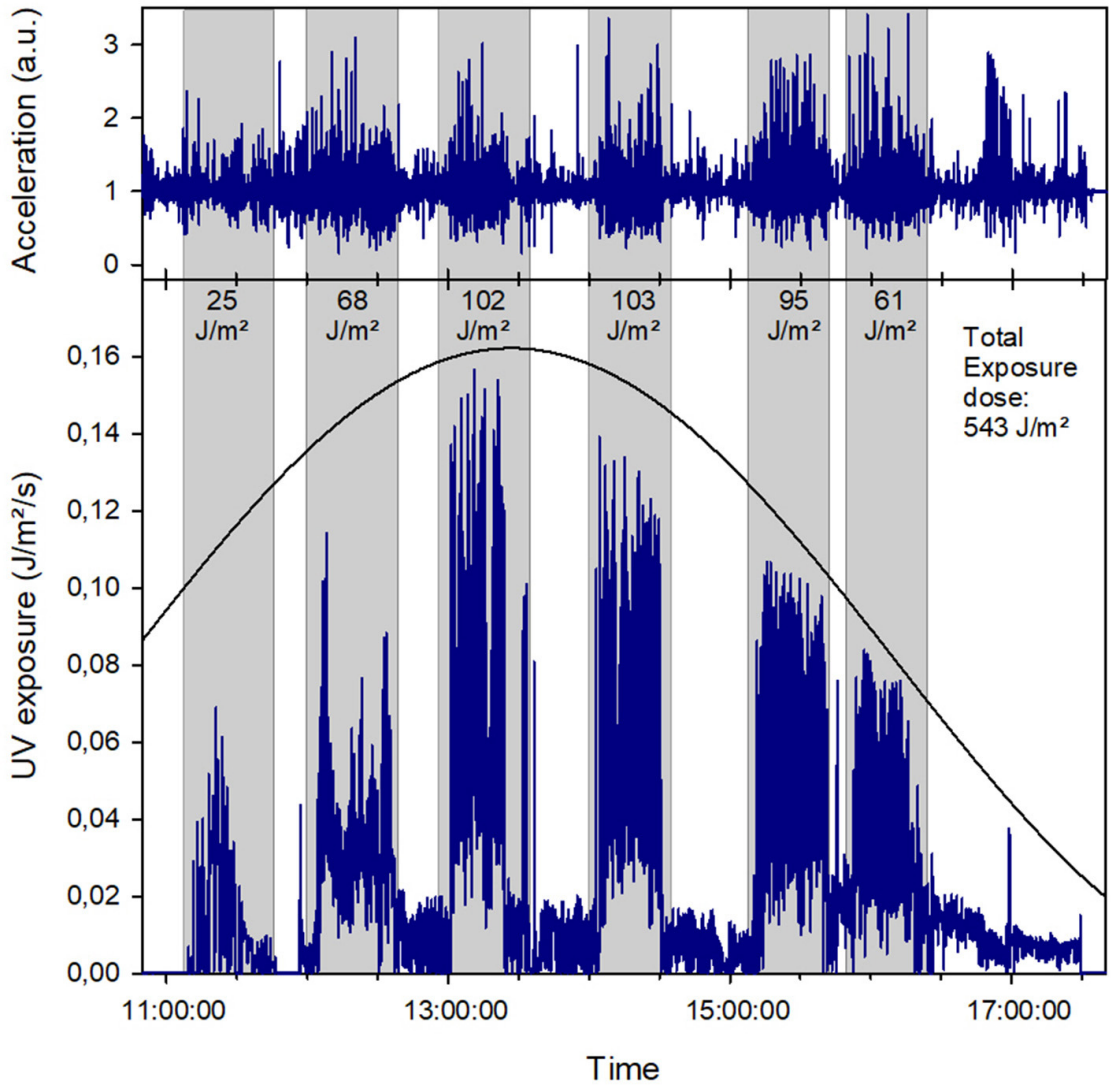
Data from a competition that went on the whole day. Six matches were played during that time (marked by gray shading). For every single game, the respective irradiance is given. Total exposure doses gives the whole day exposure. The solid line represents the daily dependence of the global irradiance (without axis, only qualitatively) Top: accelerometer data; Bottom: UV exposure.

Figure 2 shows an example of a measurement day on which one person conducted several short games in succession. It can be seen that the irradiation during the individual games follows the course of the sun and the theoretically expected daily values, ultimately culminating at noon. The individual exposure doses also increase, from 25 J/m^2^ at 11:15 a.m. to 103 J/m^2^ at 2 p.m. The total daily exposure dose is 543 J/m^2^. In this instance, the rest periods were not spent indoors but presumably in a shaded area or under a pavilion. The exposure during these times is 89 J/m^2^ in total.

### Single Day Measurements: Visiting Parks

**Figure 3:**
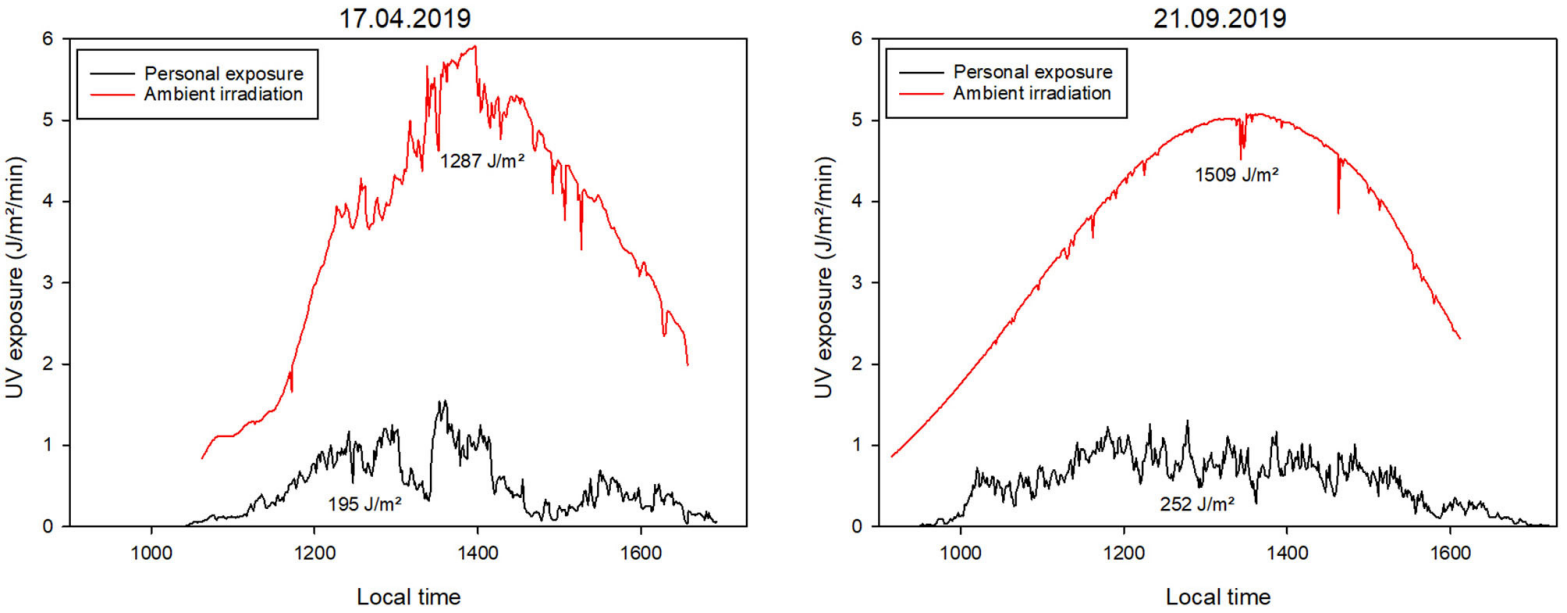
Measurement data from the “visiting parks” activity from two different days. The red curves represent the ambient radiation on each day, measured with a separate dosimeter placed horizontally on a pedestal. The black curves represent the mean value of the personal exposure calculated from the 15 individual measurements. Also provided are total UV doses for ambient and personal measurements.

On the five days of data acquisition for the “visiting parks” activity, a total of 75 measurement days were achieved (5 days times 15 dosimeters) to a total of 23,777 minutes. Figure 3 shows data acquired at “visiting parks” as the average UV radiation of all 15 volunteers per minute over the whole day for 1 day in April (17/04/2019) and 1 day in September (21/09/2021), plotted together with the ambient radiation detected by the dosimeter placed horizontally in the sun. The figure also provides information about total UV doses of ambient and personal measurements.

For the measurement day in April, a total ambient UV exposure of 1,287 J/m^2^ and an average total personal UV exposure of 195 J/m^2^ was recorded. For the measurement day in September, a total ambient UV exposure of 1,509 J/m^2^ and an average total personal UV exposure of 252 J/m^2^ was measured.

When the ambient UV radiation patterns of the different days are compared, some differences become immediately apparent. The basic shape follows the sun's path, with the sun's peak at about 1:30 p.m. The measurement in September illustrates this very well, as it was a mostly clear day with only a few clouds (can be seen as dips in the curve). The measurement in April was characterized by changeable weather, which can also be seen in the curve of the ambient UV radiation. This fluctuates much more throughout the day, as clouds of different thicknesses repeatedly shifted in front of the sun.

**Figure 4:**
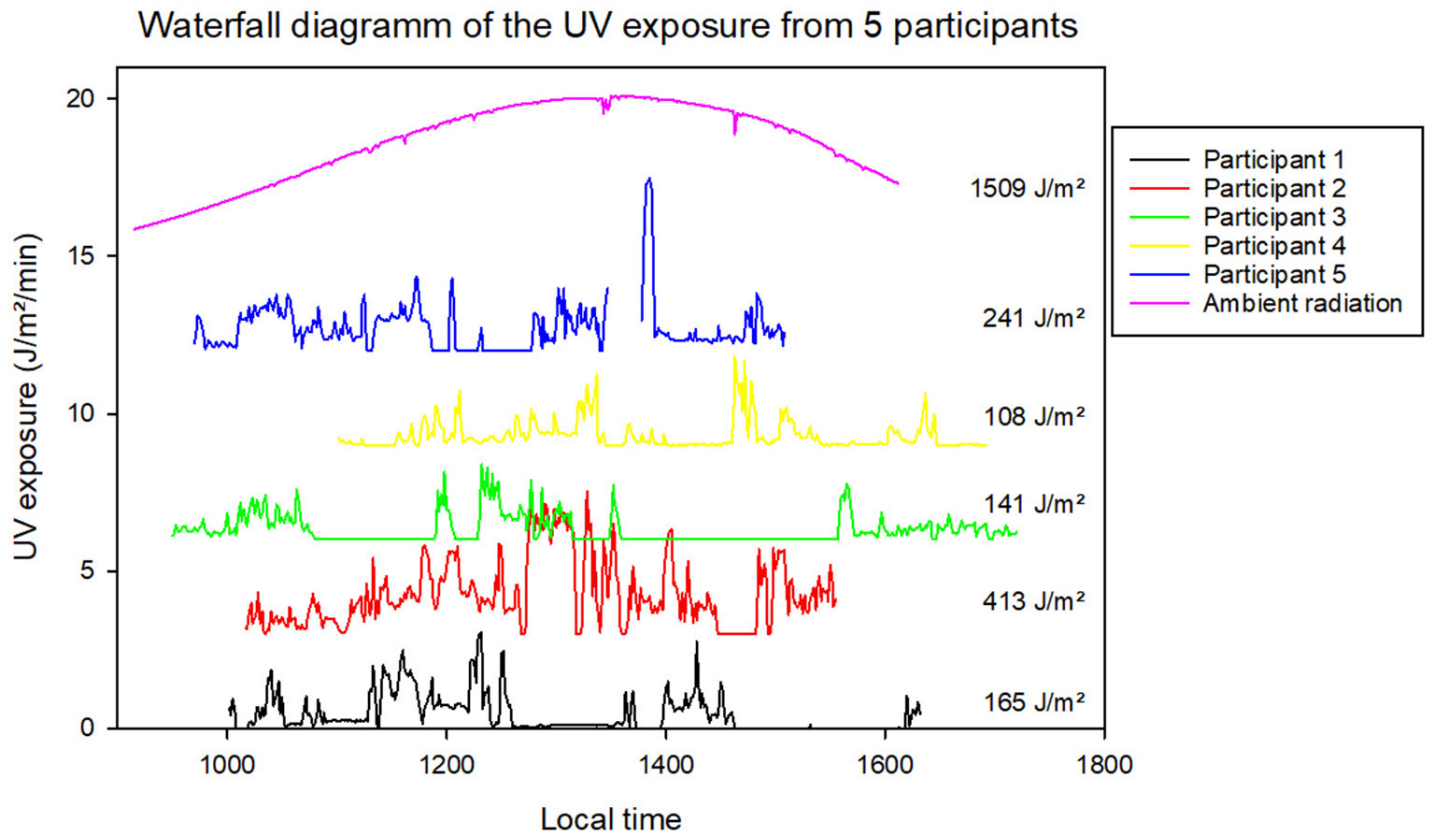
Measurement data from five selected personal UV measurements for a single day on 17/04/2019 in comparison-related ambient radiation (in magenta). Ambient radiation was measured with a separate dosimeter placed horizontally on top of a pedestal. The measurements are displayed with offsets of 3 J/m^2^/min for clarity. The total UV dose per measurement is given on the right of each graph.

The average personal UV dose for the 15 individual measurements does not follow the course of ambient radiation throughout the day. This effect can be seen more clearly when plotting some of the individual measurements separately (Figure 4). Here, the measurement data of five randomly selected volunteers were plotted in comparison to the ambient radiation. It can be seen that individual measurements vary clearly in their temporal course, measurement duration and the resulting total UV exposure. The blue curve (participant 5) is interrupted at some point. In this instance, the participant took off the dosimeter, probably during lunchtime. On the other hand, the exposure of participant 3 (green curve) is close to zero for a lot of the time. This person was probably indoors during that time but correctly did not take off the dosimeter.

### Derived Values

The resulting monthly mean exposure values per minute for both measured activities are given in Table 1.

**Table 1:** Mean UV exposure doses per minute for both activities in every month together with their standard error values and total mean values.

		**April**	**May**	**June**	**July**	**August**	**September**	**October**
Football	Mean value [J/(m^2^* min)]	-	1.02 (± 0.01)	1.17 (±0.01)	1.21 (±30.02)	0.97 (±0.02)	0.76 (±0.01)	0.39 (±0.01)
	Normalized to the dose in June	-	0.87 (±0.01)	1 (±0.01)	1.03 (±0.02)	0.83 (±0.01)	0.65 (±0.01)	0.33 (±0.01)
Visiting parks	Mean value [J/(m^2^* min)]	0.82 (±0.01)	-	0.99 (±0.02)	-	0.57 (±0.01)	0.67 (±0.01)	-
	Normalized to the dose in June	0.83 (±0.01)	-	1 (±0.02)	-	0.58 (±0.01)	0.68 (±0.01)	-
Mean ambient radiation	Normalized to radiation in June	0.54	0.81	1	0.92	0.76	0.49	0.24

*The monthly mean values are also normalized to the value in June. This was done in order to compare them to the mean ambient radiation (38) for each month, given in the last line. (“-”: no measurements during this month)*.

To compare how the individual monthly irradiation values relate to the annual cycle of irradiation by the sun, we also related these values to the solar maximum in June. The calculated values are shown in relation to the expected mean ambient radiation. The mean ambient radiation is also normalized to the maximum expected to occur in June. It can be seen that for the long-term measurements during football games, the course of accumulated UV exposure doses, on the whole, follows the course of the expected mean ambient radiation, albeit being slightly higher than expected by the fraction of the ambient level. The maximum exposure is slightly shifted, giving rise to behavioral dependence to be discussed later. Single day measurements while visiting parks can only give limited information. The tendency of the values with regard to the ambient is not clear at first glance but can be explained when taking the ambient temperature into account.

The calculation of a yearly exposure dose is rather tricky when monthly averages are to be multiplied with the time spent executing the activity while the latter is unknown. Nevertheless, knowledge of the annual dose is of particular interest when comparing different activities. As the measurements were performed from May to October, the dose has to be extrapolated to the whole year. Concluding from Supplementary Figure 1, 78% of the yearly UV exposure is accumulated during the measurement period from May to October. The missing data was then calculated by summing up the values from May to October, then dividing by 0.78. For example, if football is played for 400 min each month (games and practice), the yearly exposure calculates to 2,831 J/m^2^ (28, 3 SED). For visiting parks, a similar approach has to be chosen, taking into account that 52% of the annual exposure were covered.

Taking the approach of measuring lots of people simultaneously, determining and comparing the ERTA makes sense. Supplementary Table 1 shows the individual measurement results for the daily accumulated total UV exposure doses for all 15 volunteers for the selected dates in April and September. Two examples are given in Table 2. The exposure ratio to ambient radiation (ERTA) was calculated for each volunteer. Additionally, the mean values for total UV doses and ERTA were calculated.

**Table 2:** Total UV exposure doses for the 15 participants at measurement days in April and September.

	**17/04/2019**		**21/09/2019**	
**Volunteer #**	**Total UV dose [J/m** ^**2**^ **]**	**ERTA [%]**	**Total UV dose [J/m** ^**2**^ **]**	**ERTA [%]**
1	61	4.7	256	16.9
2	189	14.7	165	11
3	149	11.	90	6
4	111	8.6	316	20.9
5	150	11.7	321	21.3
6	124	9.6	441	29.2
7	312	24.2	413	27.4
8	294	22.8	141	9.4
9	351	27.2	184	12.2
10	201	15.6	339	22.5
11	66	5.1	157	10.4
12	299	23.2	108	7.1
13	286	22.2	220	14.6
14	241	18.7	291	19.3
15	99	7.7	342	22.7
Mean value	195 (± 24)	15.1 (± 1.9)	252 (± 28)	16.7 (± 1.9)

*Also calculated was the “Exposure Ratio to ambient radiation (ERTA)” and the corresponding mean values and standard error*.

Individual total UV exposure doses vary significantly throughout the volunteers and the same applies to the calculated ERTA values. For the measurement day in April, personal UV doses vary between 61 and 351 J/m^2^, corresponding to ERTA values between 8.8 and 37.8%. For the selected day in September, personal total UV doses vary in a similar range between 90 and 441 J/m^2^, corresponding to ERTA values between 6.3 and 33.5%. On some days, the measurement of the ambient radiation started later or ended earlier than the measurements of some of the volunteers, so the ERTA was only determined for the times when a simultaneous measurement of the ambient radiation was available.

The measurement times of the volunteers ranged between 144 and 489 min over all days. The exposure lies between 61 and 603 J/m^2^ and the ERTA is between 4.4 and 42.3%.

## Discussion

Measuring individual UV radiation exposure is both a technical and a logistical challenge. Selecting the measurement technology to be used is of central importance. Considerations in this context include the framework conditions, determined by the duration of the planned measurement campaign, as well as limitations such as the durability of the technology or the reproducibility of the results. Electronic data logger dosimeters, worn on the left upper arm, turned out to be ideal, proven systems for conducting long-term measurements of personal UV exposure (24).

Intrasystematic deviations occur when the measurement technique used is not sufficiently reliable and has a relatively large scatter. In order to arrive at suitable mean values, the use of a large number of measuring instruments is sufficient; however, an interpretation of individual results remains highly error-prone. We have followed both paths, namely the use of reliable dosimeters, as shown elsewhere (24), and the recruitment of a sufficient number of participants. Both characteristics are suitable means for long-term as well as single-day measurements to reduce deviations and uncertainties as far as possible. This is reflected by the low standard error values from descriptive statistics, as can be seen in Table 1.

Another question of equal importance is the timeframe in which the measurements should take place and what form of cooperation from the volunteers is necessary. There are two different approaches to this, each of which has both strengths and weaknesses. On the one hand, it is possible to recruit a large number of volunteers all at once at a specific place at a specific time; they then wear the dosimeters during their activities for a very limited period of less than a day. This method makes it possible to obtain a large number of measurements simultaneously in a very selective manner. Primarily, this allows direct comparison of the exposure data with regard to differences in the individual behavior of the participants since the same initial conditions (e.g., climatic) prevail for the measurements. In such cases, the volunteers' participation is based on an affect that results, for example, from being approached or being directly recruited at the measurement location. In such cases, it is advisable to only mention the requirement to wear the device within a certain period of time and specify where it is worn on the body. This was the approach taken during the visits to the garden exhibition, which resulted in daily measurements without the possibility of more precise differentiation (Table 1). The curves in Figure 4 already show that the individual behavior of the test persons must have been very different with regard to the daily routine. As previously mentioned, it is not possible to make a more detailed statement about the activities included in this period. For this, the participants would need to keep some form of a diary at the same time.

This is different when measuring a specific activity over several months. Volunteers were recruited for this purpose without them being suddenly approached. The volunteers can find out about the measurement procedure beforehand and also select which activities they wish to engage in during the measurements – in this case, “playing football.” Consequently, it is reasonable to assume that the volunteers approached the measurements with a high level of compliance, at least at the beginning. Personal support and the ease of using the measurement technology meant that this could be maintained in the majority of cases until the end of the measurement campaign. This approach gives information on UV exposure during a specific activity over the course of a year (Table 1). Even from different volunteers, individual measurements are very similar (Figure 1). The accuracy and reliability of these measurements can also be detected indirectly in the data structure. For example, in each individual measurement, two time periods of equal length can be detected, interrupted by a short time interval. These are the two half-times and the break during a football match. The pitch check required of the referee – comparable to the warming up of the players – can also be identified from the data (Figure 1 from 11:50 a.m. to 12:05 p.m.).

Each method has advantages and disadvantages, depending on the objective of the conclusion to be drawn (Table 3). Measurements over large parts of the year are advantageous because the annual course of UV radiation obviously does not follow the pattern expected from the distribution of global radiation (Table 1). Several such events must be distributed over the year to compensate for the disadvantages of single day measurements with many test persons in order to be able to make extrapolations of the course of the year with sufficient supporting points. We have used this in this study to make comparisons in different months. Ultimately, however, the activity to be studied also dictates which method is to be chosen. For a hobby such as playing football, it is relatively easy to recruit volunteers over a long period of time or to find volunteer collectives that change quickly. For visits to parks, it is easier to approach likely participants on site. When pre-selecting or recruiting volunteers, it is usually more difficult for people to predict the duration and frequency of visits of this kind throughout the year. If people are provided with measurement units for the whole year, there may be considerable periods when the units remain unused.

**Table 3:** Comparison of both approaches for measuring personal UV exposure.

**Long period measurements**	**Single day measurements**
Every participant has a dosimeter for several months	Participants wear the dosimeter only for a couple of hours
Participants need to read out the dosimeters and remember wearing it for the specified activity	No technical effort for the participant
Data gives strong statistics over long periods, average of weather and other environmental conditions is included in dataset	Very good statistics on single days in a specific location, but to contextualize these measurements to the course of a whole year more effort is needed (weather data, ambient measurement, etc.)
Long-term behavioral differences between people visible	Direct comparison of different people's behavior is possible
Data suitable for legal discussions and prevention conceptualization	Combinable with awareness campaigns of different stakeholders
Suitable for long-term or repeated intervention studies	Suitable for short-term or single-shot intervention studies

It is better to choose long-term measurements to derive an annual exposure value or the mean value over a longer period of time, as the behavior of test persons and the influences of secondary parameters such as weather can be better controlled. Measurement of personal UV exposure is largely affected by the personal behavior of participants (39, 40). Environmental and behavioral factors were both important in determining overall levels of exposure and distribution by site (33). For example, personal exposure is strongly affected by season (27). In the case of temperature, personal exposure increases first but seems to go down after a specific temperature is exceeded (41, 42). For leisure time settings, the fair-weather effect has to be named as probably one of the most determining factors: as in occupational settings, people working outdoors seldom have a chance to have an impact on outdoor exposure, it can be seen that during leisure time, people prefer to be outdoors when there is good weather. That is because leisure time exposure is consequently higher on average and, in many cases, higher than expected. In order to address this effect, we compared weather-related ambient UV levels to personal UV exposure levels by means of the ERTA. For this purpose, it makes sense to record ambient UV radiation, e.g. using another dosimeter in parallel to the personal measurements.

**Figure 5:**
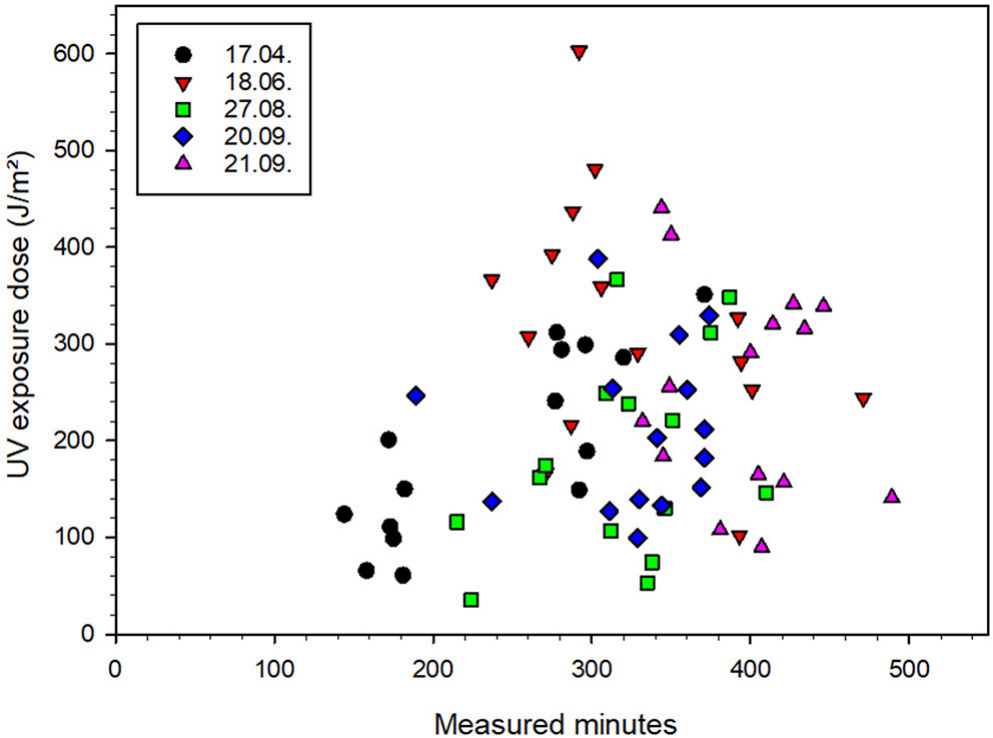
UV exposure doses and total measured minutes for all participants of the “visiting parks” activity. The different measurement days are color coded, with 15 participants for each day. Each participant represents one data point. As an example, the red triangle on top represents a participant who took part on 18^th^ of June, wore the dosimeter for 292 min and received an exposure dose of 603 J/m^2^.

As reported, a person's exposure ratio to ambient (ERTA) depends on the time spent outdoors and ranges between 3 and 5% on an annual average, but up to 30% during outdoor episodes (18). Our studies can confirm this (Table 2); only in 6 out of 75 cases the ratio of 30% is slightly exceeded (Supplementary Table 1). For the visit to the garden exhibition, no statistically significant correlation between the measurement time and the irradiation (dose) can be found (Figure 5). This clearly indicates that the behavior of people during visiting parks can differ significantly from person to person and thus lead to very individual patterns of exposure. In this respect, the approach of selectively measuring a large number of people for activities such as visiting parks, which ultimately comprise many smaller individual activities, is an appropriate choice to address this state of affairs. This is not the case for sporting events such as football, where a correlation can be found between measurement time or time spent outdoors and exposure (Figure 5). As a result, this indicates that these are typical exposure patterns for the activity.

This study, however, also provides further insights into the chosen activities over and above the methodological analysis, which can be of particular relevance as regards the field of prevention. It is evident that high UV irradiation levels can be acquired while playing a game of football, even if exposure times are short. Since people usually play wearing short clothing and often without headgear, the cumulative impact on the risk of skin cancer can be considerable. According to Fitzpatrick (43), any of the UV exposures identified are sufficient to cause sunburn, especially in fair skin types. The mean values given in Table 1 can be used effectively to determine individual irradiation levels. An Australian study can be found in an international comparison (28). The irradiation of 99 J/(m^2^*h) to 140 J/(m^2^*h) measured in Hervey Bay (Australia, 25 °S) can be converted to German latitudes using latitude factors (11). Using a latitude factor of 2.4, a minute value of 0.69 J/(m^2^*min) to 0.96 J/(m^2^*min) is calculated. This agrees very well-with our values within the error limits.

Although there is still no legally binding exposure limit value for UV exposure, let alone concrete legal policy plans, it is well-worth comparing it with the exposure limit values proposed by the scientific community. The World Health Organization (WHO) and the International Commission on non-Ionizing Radiation Protection (ICNIRP) (44) recommend a maximum daily exposure of 1 SED [1 SED = 100 J/m^2^ erythema-weighted irradiation; incoming radiation weighted with the erythema action spectrum S_er_ from CIE (35)], which is about half to two-thirds of a sunburn dose for the vulnerable skin type I according to Fitzpatrick. In summer, in particular, this irradiation is reached very quickly if a person is active outdoors during the time of the highest exposures from 11 a.m. to 3 p.m. Sun protection measures should therefore also be taken into account for leisure time activities. This appears to be easier for activities in parks, as it is easier to install some forms of shade than, for example, on a football pitch. In the case of the latter, measures of prevention must be discussed with the football associations, the use of adapted clothing or sunscreen, which is suitable for the workplace and has been tested for employees who sweat heavily, appears to be individually possible and advisable.

This study has limitations. As the data set was recorded in Germany, checking the transferability to other countries and customs is necessary. The closer one gets to the equator, the stronger the UV irradiation becomes. As a result, the measured exposure values can vary, sometimes significantly, when measurements are taken in other countries or areas of the world. It should also be noted that the results obtained are, in principle, subject to the well-known problems of personal dosimetry but these have been primarily countered by using a large number of participants and a large number of data sets and validation methods. The most significant problems and inaccuracies were caused by the volunteers wearing the dosimeter incorrectly or putting the dosimeter down during measurements. The latter could be detected and corrected by also taking into account the measured values of the acceleration sensor integrated into the dosimeter. However, it is not possible to completely exclude errors due to incorrectly wearing the dosimeter. In the case of movements that are somewhat random with respect to the orientation to the sun, we expect the incorrect wearing of the dosimeter to have only a small effect on the data, provided that the measurement time is sufficiently long. It is important to ensure that the volunteers are thoroughly instructed and supervised when conducting such studies. Another factor to consider is the possibility that the volunteers' compliance may decrease, especially if the study is conducted over a longer period of time. However, this does not necessarily lead to poorer data quality but only to a possibly lower number of measured values. Again, for this project to be successful, it is essential to provide personal supervision and contact, not only at the beginning and end of the measurements but also throughout the whole course of the project.

Skin cancer caused by UV radiation continues to be a major issue, both in the occupational and leisure spheres. Our measurements have shown that in the recreational sector, considerable UV exposure doses can be reached even after a short period of time, which ultimately contribute to chronic light damage to the skin. It is important to counter this, firstly by measuring outdoor activities consistently as a basis for developing individual prevention approaches, which in turn provides evidence of the existence of the risk, and secondly by raising awareness, also by means of local events that provide information and measurements of individual exposure. This study serves both of these objectives and serves as a model for future measurements with related questions.

## Data Availability Statement

The original contributions presented in the study are included in the article/Supplementary Material, further inquiries can be directed to the corresponding author/s.

## Ethics Statement

Ethical review and approval was not required for the study on human participants in accordance with the local legislation and institutional requirements. The patients/participants provided their written informed consent to participate in this study.

## Author Contributions

TH and MW: conceptualisation and project administration. TH, CS, and MW: validation and writing – original draft preparation. TH: data curation and investigation. MW: writing – review, editing, and supervision. All authors have read and agreed to the published version of the manuscript.

## Funding

The funding of this project was institutional.

## Conflict of Interest

The authors declare that the research was conducted in the absence of any commercial or financial relationships that could be construed as a potential conflict of interest.

## Publisher's Note

All claims expressed in this article are solely those of the authors and do not necessarily represent those of their affiliated organizations, or those of the publisher, the editors and the reviewers. Any product that may be evaluated in this article, or claim that may be made by its manufacturer, is not guaranteed or endorsed by the publisher.

## Supplementary Material

Supplementary Figure 1 Theoretical values for seasonal and daily dependence of UVR global irradiance. (a) Seasonal factors for the global UV radiation for every month throughout the year (northern hemisphere). The factors for the southern hemisphere are shifted by 6 months. (b) Daytime factors for the global UV radiation throughout a day in temperate climate during summertime. Data taken from (11).

## References

[1] International Agency for Research on Cancer. Solar and Ultra-Violet Radiation. Lyon: IARC (1992).

[2] Young AR Morgan KA Harrison GI Lawrence KP Petersen B Wulf HC. A revised action spectrum for vitamin D synthesis by suberythemal UV radiation exposure in humans in *vivo*. PNAS. (2021) 118:e2015867118. 10.1073/pnas.2015867118 34580202 PMC8501902

[3] MacLaughlin JA Anderson RR Holick MF. Spectral character of sunlight modulates photosynthesis of previtamin D3 and its photoisomers in human skin. Science. (1982) 216:1001–3. 10.1126/science.6281884 6281884

[4] Vitasa BCT Strickland HR Rosenthal PT West FS Abbey S Ket Ng H. Association of non-meloma skin cancer and actinic keratosis with cumulative solar ultraviolet exposure in Maryland watermen. Cancer. (1990) 65:2811–7. 10.1002/1097-0142(19900615)65:12<2811::AID-CNCR2820651234>3.0.CO;2-U 2340474

[5] Kricker A Armstrong BK English DR Heenan PJ. Does intermittent sun exposure cause basal cell carcinoma? A case-control study in Western Australia. Int J Cancer. (1995) 60:489–94. 10.1002/ijc.2910600411 7829262

[6] Dirschka T Pellacani G Micali G Malvehy J Stratigos AJ Casari A. A proposed scoring system for assessing the severity of actinic keratosis on the head: actinic keratosis area and severity index. J Eur Acad Dermatol Venereol. (2017) 31:1295–302. 10.1111/jdv.14267 28401585

[7] Dreno B Cerio R Dirschka T Nart IF Lear JT Peris K. A novel actinic keratosis field assessment scale for grading actinic keratosis disease severity. Acta dermato-venereologica. (2017) 97:1108–13. 10.2340/00015555-2710 28536731

[8] Robert-Koch-Institut. Bericht zum Krebsgeschehen in Deutschland: Bundesministerium für Gesundheit. Berlin (2016).

[9] Wittlich M. Criteria for occupational health prevention for solar uvr exposed outdoor workers-prevalence, affected parties, and occupational disease. Front Public Health. (2022) 9:2290. 10.3389/fpubh.2021.772290 35155340 PMC8826221

[10] Wittlich M Westerhausen S Strehl B Schmitz M Stöppelmann W Versteeg H. IFA Report 4/2020 - Exposition von Beschäftigten gegenüber solarer UV. Strahlung: Ergebnisse des Projekts mit Genesis-UV. Deutsche Gesetzliche Unfallversicherung e.V. (DGUV), Berlin: Germany (2020).

[11] Wittlich M Westerhausen S Kleinespel P Rifer G Stoppelmann W. An approximation of occupational lifetime UVR exposure: algorithm for retrospective assessment and current measurements. J Eur Acad Dermatol Venereol. (2016) 30(Suppl 3):27–33. 10.1111/jdv.13607 26995020

[12] Thieden E Collins SM Philipsen PA Murphy GM Wulf HC. Ultraviolet exposure patterns of Irish and Danish gardeners during work and leisure. Br J Dermatol. (2005) 153:795–801. 10.1111/j.1365-2133.2005.06797.x 16181463

[13] Schneider S Diehl K Schilling L Spengler M Greinert R Gorig T. Occupational UV exposure and sun-protective behaviour in german outdoor workers: results of a nationwide study. J Occup Environ Med. (2018) 60:961–7. 10.1097/JOM.0000000000001397 30020216

[14] Schilling L Schneider S Gorig T Spengler M Greinert R Breitbart EW. “Lost in the sun”-The key role of perceived workplace support for sun-protective behavior in outdoor workers. Am J Ind Med. (2018) 61:929–38. 10.1002/ajim.22905 30175492

[15] Radespiel-Tröger M. Berufliche UV-Belastung und Hautkrebs. Zbl Arbeitsmed. (2011) 61:112–25. 10.1007/BF03346247

[16] Grandahl K Eriksen P Ibler KS Bonde JP Mortensen OS. Measurements of solar ultraviolet radiation exposure at work and at leisure in Danish workers. Photochem Photobiol. (2018) 94:807–14. 10.1111/php.12920 29603236

[17] Herlihy E Gies PH Roy CR Jones M. Personal dosimetry of solar uv radiation for different outdoor activities. Photochem Photobiol. (1994) 60:288–194. 10.1111/j.1751-1097.1994.tb05106.x 7972383

[18] Siani AM Casale GR Diémoz H Agnesod G Kimlin MG Lang CA. Personal UV exposure in high albedo alpine sites. Atmosc Chem Phy. (2008) 8:3749–60. 10.5194/acp-8-3749-2008

[19] Knuschke P Barth J. Biologically weighted personal UV dosimetry. J Photochem Photobiol. (1996) 36:77–83. 10.1016/1011-1344(95)07223-3 8988614

[20] Serrano MA Cañada J Moreno JC Gurrea G. Personal UV exposure for different outdoor sports. Photochem Photobiol Sci. (2014)) 13:671–9. 10.1039/C3PP50348H 24535504

[21] Wittlich M John SM Tiplica GS Sălăvăstru CM Butacu AI Modenese A. Personal solar ultraviolet radiation dosimetry in an occupational setting across Europe. J Eur Acad Dermatol Venereol. (2020) 34:1835–41. 10.1111/jdv.16303 32080895

[22] Thieden E Philipsen PA Heydenreich J Wulf HC, UV. Radiation exposure related to age, sex, occupation, and sun behavior based on time-stamped personal dosimeter readings. Arch Dermatol. (2004) 140:197–203. 10.1001/archderm.140.2.197 14967793

[23] Cockell CS Scherer K Horneck G Rettberg P Facius R Gugg-Helminger A. Exposure of arctic field scientists to ultraviolet radiation evaluated using personal dosimeters. Photochem Photobiol. (2007) 74:570–8. 10.1562/0031-8655(2001)0740570EOAFST2.0.CO2 11683037

[24] Strehl C Heepenstrick T Knuschke P Wittlich M. Bringing light into darkness-comparison of different personal dosimeters for assessment of solar ultraviolet exposure. Int J Environ Res Public Health. (2021) 18:9071. 10.3390/ijerph18179071 34501660 PMC8431201

[25] Schmalwieser AW Siani AM. Review on non-occupational personal solar UV exposure measurements. Photochem Photobiol. (2018) 94:900–15. 10.1111/php.12946 29856894

[26] Siani AM Casale GR Sisto R Borra M Kimlin MG Lang CA. Short-term UV exposure of sunbathers at a Mediterranean Sea site. Photochem Photobiol. (2009) 85:171–7. 10.1111/j.1751-1097.2008.00413.x 18713135

[27] Sun J Lucas RM Harrison S van der Mei I Armstrong BK Nowak M. The relationship between ambient ultraviolet radiation (UVR) and objectively measured personal UVR exposure dose is modified by season and latitude. Photochem Photobiol Sci. (2014) 13:1711–8. 10.1039/C4PP00322E 25311529

[28] Downs NJ Parisi AV. Patterns in the received facial UV exposure of school children measured at a subtropical latitude. Photochem Photobiol. (2007) 84:90–100. 10.1039/B607553C 18173708

[29] Cadet JM Bencherif H Cadet N Lamy K Portafaix T Belus M. Solar UV radiation in the tropics: human exposure at reunion Island (21 degrees S, 55 degrees E) during summer outdoor activities. Int J Environ Res Public Health. (2020) 17:2767. 10.3390/ijerph17218105 33153111 PMC7662767

[30] Moehrle M. Ultraviolet exposure in the Ironman triathlon. Med Sci Sports Exerc. (2001) 33:1385–6. 10.1097/00005768-200108000-00021 11474342

[31] Moehrle M Heinrich L Schmid A Garbe C. Extreme UV exposure of professional cyclists. Dermatology. (2000) 201:44–5. 10.1159/000018428 10971059

[32] Serrano MA Cañada J Moreno JC. Erythemal ultraviolet exposure of cyclists in valencia, Spain. Photochem Photobiol. (2010) 86:716–21. 10.1111/j.1751-1097.2009.00693.x 20158673

[33] Holman CDJ Gibson IM Stephenson M Armstrong BK. Ultraviolet irradiation of human body sites in relation to occupation and outdoor activity: field studies using personal UVR dosimeters. Clin Exp Dermatol. (1983) 8:269–77. 10.1111/j.1365-2230.1983.tb01779.x 6883792

[34] Nurse V Wright CY Allen M McKenzie RL. Solar Ultraviolet radiation exposure of South African marathon runners during competition marathon runs and training sessions: a feasibility study. Photochem Photobiol. (2015) 91:971–9. 10.1111/php.12461 25918823

[35] International Commission on Illumination (CIE). ISO/CIE 17166:2019(E): Erythema Reference Action Spectrum And Standard Erythema Dose. ISO/TC 274, Light and lighting, International Commission on Illumination (CIE). Vienna (2019). p. 12.

[36] Godar DE, UV. Doses Worldwide. Photochem Photobiol. (2005) 81:736–49. 10.1562/2004-09-07-IR-308R.1 15819599

[37] Knuschke P Unverricht I Ott G Janßen M. Personenbezogene Messung der UV-Exposition von Arbeitnehmern im Freien. Dortmund: Bundesanstalt für Arbeitsschutz und Arbeitsmedizin. (2007).

[38] Knuschke P Bauer A Mersiowsky K Püschel A Rönsch H Ott G. Schutzkomponenten bei solarer UV-Exposition. Bundesanstalt für Arbeitsschutz und Arbeitsmedizin. (2015).

[39] Diffey BL. Time and Place as Modifiers of Personal UV Exposure. Int J Environ Res Public Health. (2018) 15:1112. 10.3390/ijerph15061112 29848972 PMC6025391

[40] Petersen B Triguero-Mas M Maier B Thieden E Philipsen PA Heydenreich J. Sun behaviour and personal UVR exposure among Europeans on short term holidays. J Photochem Photobiol. (2015) 151:264–9. 10.1016/j.jphotobiol.2015.08.022 26332747

[41] Cahoon EK Wheeler DC Kimlin MG Kwok RK Alexander BH Little MP. Individual, environmental, and meteorological predictors of daily personal ultraviolet radiation exposure measurements in a United States cohort study. PLoS ONE. (2013) 8:e54983. 10.1371/journal.pone.0054983 23405102 PMC3566166

[42] Xiang F Harrison S Nowak M Kimlin M van der Mei I Neale RE. Weekend personal ultraviolet radiation exposure in four cities in Australia: influence of temperature, humidity and ambient ultraviolet radiation. J Photochem Photobiol. (2015) 143:74–81. 10.1016/j.jphotobiol.2014.12.029 25600266

[43] Fitzpatrick TB. The validity and practicality of sun-reactive skin types I through VI. Arch Dermatol. (1988) 124:869–71. 10.1001/archderm.124.6.869 3377516

[44] Protection ICoN-IR. ICNIRP statement-Protection of workers against ultraviolet radiation. Health Physics. (2010) 99:66–87. 10.1097/HP.0b013e3181d85908 20539126

