# Supplementary material for: Probing Different Approaches in Ultraviolet Radiation Personal Dosimetry – Ball Sports and Visiting Parks
Source: Front Public Health. 2022 Apr 27;10:868853. doi: 10.3389/fpubh.2022.868853 (PMC9092294; doi:10.3389/fpubh.2022.868853)
Supplement: Supplementary file 1 [file Table_1.pdf]

Supplementary Table 1:

Total UV doses for the 15 participants on all measurement days. Also calculated was the "Exposure Ratio to ambient radiation (ERTA)" and the corresponding mean values and standard error. Since the ambient measurements in some cases started later or ended earlier than some measurements from the participants, the ERTA is only calculated for times where a corresponding ambient measurement was available.

	17/04/2019			18/06/2019			27/08/2019			20/09/2019			21/09/2019		
Volun-teer #	measured minutes	Total UV dose (J/m²)	ERTA (%)	measured minutes	Total UV dose (J/m²)	ERTA (%)	measured minutes	Total UV dose (J/m²)	ERTA (%)	measured minutes	Total UV dose (J/m²)	ERTA (%)	measured minutes	Total UV dose (J/m²)	ERTA (%)
1	181	61	8.8	287	216	12.0	267	162	14.9	311	127	11.7	349	256	19.2
2	297	189	17.1	292	603	32.5	215	116	12.0	237	137	16.4	405	165	11.5
3	292	149	13.6	271	166	9.7	351	221	14.4	329	99	8.7	407	90	6.3
4	173	111	16.6	393	102	4.4	338	74	4.8	313	254	23.1	434	316	22.2
5	182	150	21.8	329	291	14.5	271	175	15.8	360	253	20.1	414	321	22.8
6	144	124	37.8	392	327	14.1	387	349	20.4	355	310	24.8	344	441	33.5
7	278	312	29.8	394	282	12.5	312	106	7.7	371	212	16.6	350	413	31.0
8	281	294	27.8	260	307	18.5	323	238	16.7	371	182	14.6	489	141	9.5
9	371	351	26.9	306	359	18.9	316	367	26.3	189	246	42.3	345	184	14.6

	17/04/2019			18/06/2019			27/08/2019			20/09/2019			21/09/2019		
Volun-teer #	measured minutes	Total UV dose (J/m²)	ERTA (%)	measured minutes	Total UV dose (J/m²)	ERTA (%)	measured minutes	Total UV dose (J/m²)	ERTA (%)	measured minutes	Total UV dose (J/m²)	ERTA (%)	measured minutes	Total UV dose (J/m²)	ERTA (%)
10	172	201	30.5	237	366	23.9	346	130	8.2	369	152	12.2	446	339	23.3
11	158	66	17.0	401	253	10.7	410	146	8.0	374	330	26.4	421	157	10.8
12	296	299	22.2	471	244	10.3	224	36	3.1	344	133	11.5	381	108	8.3
13	320	286	23.0	302	481	25.5	375	312	18.5	304	388	34.1	332	220	18.6
14	277	241	22.9	275	392	22.4	335	53	3.5	341	203	17.8	400	291	20.2
15	175	99	14.7	288	437	24.2	309	249	16.9	330	140	12.2	427	342	23.5
Mean value	240	195	22	327	322	16.9	319	182	12.7	327	211	19.5	396	252	18.4
Std Error	18	24	1.9	17	32	1.9	14	26	1.7	13	22	2.3	11	28	2.0
