# Supplementary figures and images for: Probing Different Approaches in Ultraviolet Radiation Personal Dosimetry – Ball Sports and Visiting Parks
Source: Front Public Health. 2022 Apr 27;10:868853. doi: 10.3389/fpubh.2022.868853 (PMC9092294; doi:10.3389/fpubh.2022.868853)

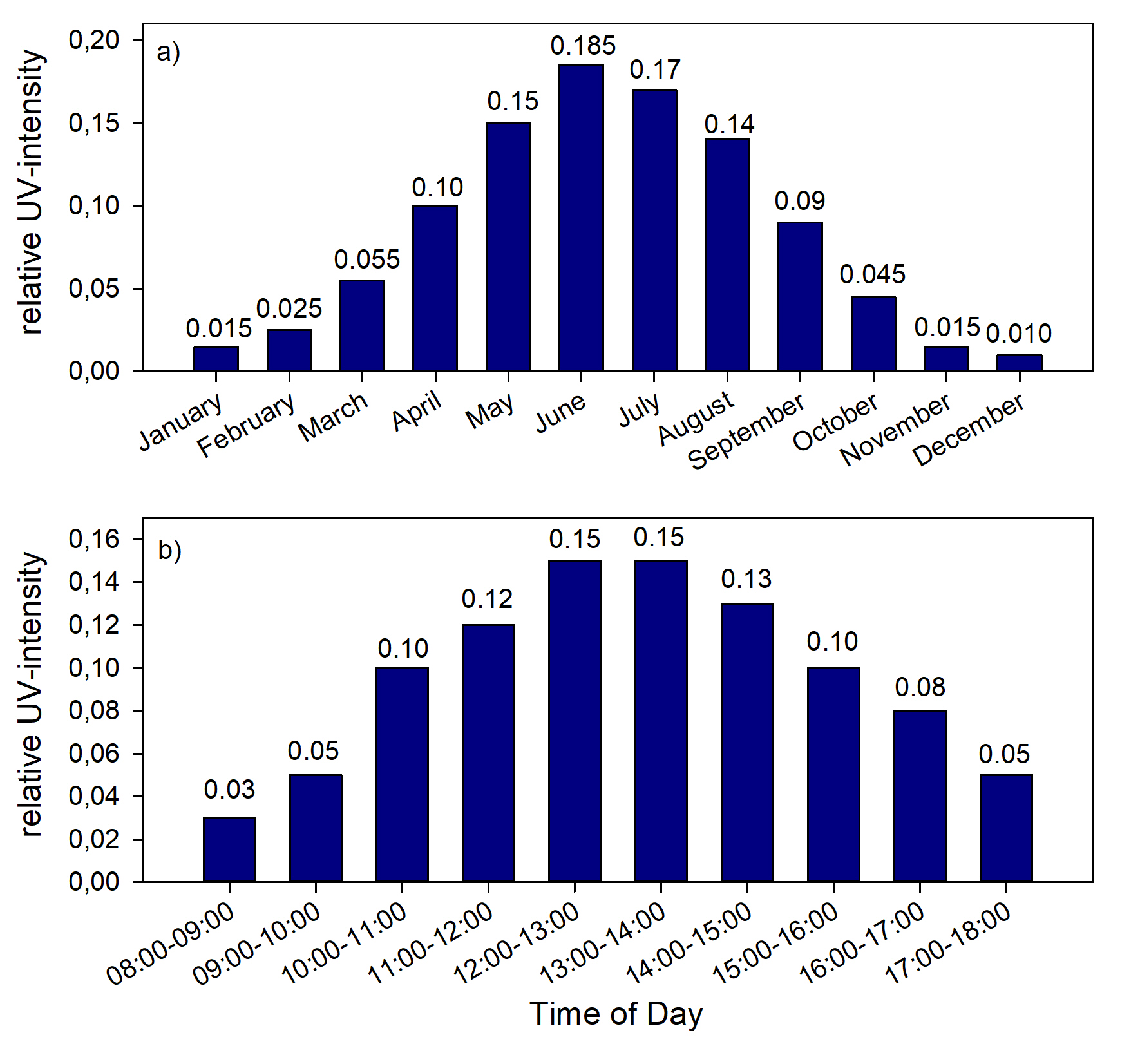

Supplement: Supplementary Figure 1 — Theoretical values for seasonal and daily dependence of UVR global irradiance. (a) Seasonal factors for the global UV radiation for every month throughout the year (northern hemisphere). The factors for the southern hemisphere are shifted by 6 months. (b) Daytime factors for the global UV radiation throughout a day in temperate climate during summertime. Data taken from (11). [file Image_1.JPEG]
